# Origins of amyloid-β

**DOI:** 10.1186/1471-2164-14-290

**Published:** 2013-04-30

**Authors:** William G Tharp, Indra Neil Sarkar

**Affiliations:** 1 Center for Clinical and Translational Science, University of Vermont, Given Courtyard N309, 89 Beaumont Avenue, Burlington, VT, 05405, USA; 2 Division of Endocrinology, Department of Medicine, University of Vermont, Given Courtyard N309, 89 Beaumont Avenue, Burlington, VT, 05405, USA; 3 Department of Microbiology and Molecular Genetics, University of Vermont, Given Courtyard N309, 89 Beaumont Avenue, Burlington, VT, 05405, USA; 4 Department of Computer Science, University of Vermont, Given Courtyard N309, 89 Beaumont Avenue, Burlington, VT, 05405, USA

**Keywords:** Amyloid, Alzheimer disease, Phylogenetics, In silico, Aggregation, Maximum parsimony, Bayesian inference

## Abstract

**Background:**

Amyloid-β plaques are a defining characteristic of Alzheimer Disease. However, Amyloid-β deposition is also found in other forms of dementia and in non-pathological contexts. Amyloid-β deposition is variable among vertebrate species and the evolutionary emergence of the amyloidogenic property is currently unknown. Evolutionary persistence of a pathological peptide sequence may depend on the functions of the precursor gene, conservation or mutation of nucleotides or peptide domains within the precursor gene, or a species-specific physiological environment.

**Results:**

In this study, we asked when amyloidogenic Amyloid-β first arose using phylogenetic trees constructed for the Amyloid-β Precursor Protein gene family and by modeling the potential for Amyloid-β aggregation across species *in silico*. We collected the most comprehensive set of sequences for the Amyloid-β Precursor Protein family using an automated, iterative meta-database search and constructed a highly resolved phylogeny. The analysis revealed that the ancestral gene for invertebrate and vertebrate Amyloid-β Precursor Protein gene families arose around metazoic speciation during the Ediacaran period. Synapomorphic frequencies found domain-specific conservation of sequence. Analyses of aggregation potential showed that potentially amyloidogenic sequences are a ubiquitous feature of vertebrate Amyloid-β Precursor Protein but are also found in echinoderm, nematode, and cephalochordate, and hymenoptera species homologues.

**Conclusions:**

The Amyloid-β Precursor Protein gene is ancient and highly conserved. The amyloid forming Amyloid-β domains may have been present in early deuterostomes, but more recent mutations appear to have resulted in potentially unrelated amyoid forming sequences. Our results further highlight that the species-specific physiological environment is as critical to Amyloid-β formation as the peptide sequence.

## Background

The Amyloid-β Precursor Protein (AβPP, APP) has been intensively studied due to its role in the generation of pathogenic cortical plaques in Alzheimer Disease [1]. It belongs to a gene family with deep evolutionary origins and is a member of a highly conserved protein family of type-1 transmembrane proteins [2-4]. The AβPP family consists of up to three homologues in vertebrate species: AβPP, amyloid precursor like protein 1 (APLP-1), and amyloid precursor like protein 2 (APLP-2). Invertebrate species genomes encode a single homologue referred to as either amyloid precursor like 1 protein (APL-1) or AβPP-like 1 protein (APPL-1). Vertebrates and flatworms exhibit ubiquitous expression of at least one member of the AβPP family, while fruit flies express APPL-1 only in neurons. In all species, AβPP proteins are cleaved into multiple peptides and fragments by a series of proteases, but only vertebrate AβPP contains the sequence coding the pathological Amyloid-β (Aβ) peptide fragment.

The β-fold intrinsic to amyloid formation is a commonly observed biochemical property [5-7]. Amyloid formation is observed in non-pathological contexts from an efficient steric mechanism for storage of small peptide hormones to rudimentary forms of biological compartmentalization [5,8]. The neuropathological changes observed in the brains of patients with Alzheimer Disease led to the formation of the Amyloid Hypothesis, which implicates both extracellular deposits of Aβ fibrils and low-order intracellular Aβ oligomers in the disruption of neuronal function, distortion of neural architecture, and induction of inflammation [1].

Mutations in the AβPP sequence and in associated proteases have been independently associated with familial early onset Alzheimer Disease characterized by rapidly progressive dementia and heavy Aβ plaque burden [9]. Recently, a protective mutation in AβPP reducing the formation of Aβ was identified [10]. However, >95% of sporadic Alzheimer Disease exhibits no mutation in the AβPP gene sequence. Further, deposition of Aβ is not limited to Alzheimer Disease. Aβ plaques have been observed in vascular dementias, Lewy body dementia, and Parkinson Disease with dementia, as well as in the brains of aged individuals without any cognitive deficits [11-14]. Together, these studies indicate that while the sequence of Aβ can contribute to the progression and severity of disease factors regulating the production by proteolysis and the degradation and clearance of Aβ, it also plays a critical role in generation of Aβ pathology.

Beyond the eponymous production of Aβ, AβPP processing produces other active peptides with functions ranging from hemostatic modulators to trophic factors to pro-apoptotic proteins [15-18]. There is a substantial body of knowledge focusing on the neural impacts of AβPP and Aβ. However, this family of proteins is also widely expressed in peripheral tissues of vertebrate species including skin, skeletal muscle, leukocytes, platelets, intestinal epithelia, pancreas, and adipose tissue. The function and regulation of non-neuronal AβPP are not fully understood [19-26].

The AβPP family is variably essential for viability among species. Experimental data show that the N-terminus of APL-1 is necessary for progression through molting stages by nematodes [27]. The C-terminus of at least one member of the AβPP family is necessary for viability in early parturition of knockout mouse models [28-30]. Drosophila models without APPL-1 show subtle neuronal patterning defects but remain viable and able to reproduce [31]. Zebrafish knockout models have impaired body development and synaptogenesis [32,33]. Each of these models can be rescued by expression of truncated portions of AβPPs, indicating that absences of different domains are responsible for the observed lethality or defects in each model. Thus, the persistence of this protein family appears domain-dependent among species despite high evolutionary conservation of the entire gene.

**Figure 1:**
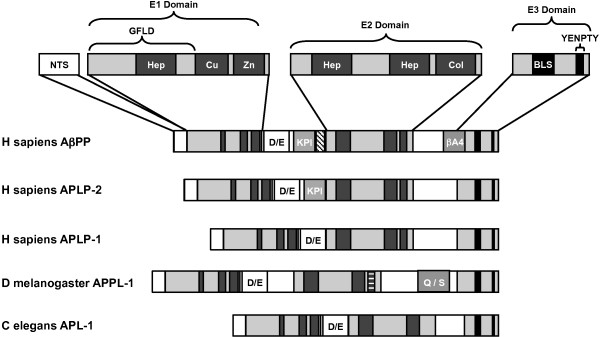
**Conserved Regions of the Amyloid-β Precursor Protein Gene Family.** Schematic representations of the five members of the Amyloid-β Precursor Protein (AβPP) gene family show multiple conserved domains: N-Terminal Signal peptide (NTS), growth factor-like domain (GFLD), heparin binding domain (Hep), copper binding domain (Cu), zinc binding domain (Zn), an acidic amino-acid rich region (D/E), collagen binding domain (Col), a basolateral sorting signal (BLS), and a clathrin-binding internalization signal domain (YENPTY). Certain members of the gene family also contain a Kunitz-protease inhibitor domain (KPI), the amyloid-β forming region (βA4), an OX-2 domain (diagonal hashmarks), a putative collagen binding domain (horizontal white hashmarks), and/or a glutamine and serine-rich region (Q/S).

The major conserved regions found in AβPP family proteins include two ectoplasmic domains (E1 and E2), which contain extracellular matrix and divalent cation binding regions and a growth-factor-like domain (GFLD), and the cytoplasmic region (E3) that contains a basolateral sorting signal (BLS) and an NPXY internalization sequence (YENPTY) (Figure 1). The corresponding nucleic acids coding the domains are termed D1, D2, and D3, respectively. Other important conserved domains include a Kunitz-protease inhibitor (KPI) domain found only in vertebrate AβPP and APLP-2, as well as the βA4 region that gives rise to Aβ in certain vertebrate species. Interestingly, it has been demonstrated that the corresponding region of APPL-1 in Drosophila melanogaster can form amyloid deposits when co-expressed in high levels with the Drosophila β-secretase [34]. Similar to the conservation of the E1, E2, and E3 domains, the βA4 region and corresponding regions may have arisen from a common ancestral domain [35]. It is not known when the amyloidogenic trait first appeared in this gene family nor why species with nearly identical Aβ sequences do not develop Aβ deposits.

Previous phylogenetic studies showed that this ancient protein family has been widely distributed among multicellular eukaryotes since at least the divergence of protostomia and deuterostomia [36]. These studies and corresponding conclusions are based on at most ten sequences that were trimmed and concatenated, focusing solely on the major conserved domains (D1, D2, and D3 only). Use of trimmed sequences does yield cleaner sequence alignments and better branch supports on the phylogenetic tree, but ignores potentially valuable evolutionary data encoded in adjoining regions. For the AβPP gene family in particular, the omission of Aβ from the analyses occludes an understanding of the evolution of the pathological eponymous domain. Despite wide distribution of the AβPP family across species, it is not know when amyloidogenic Aβ peptides first evolved. This study uses the full complement of available molecular sequence data to provide an *in silico* model of the evolutionary history of this essential gene family and the origin of the Aβ peptide.

## Results

### Phylogenetics of AβPP gene family

Amino acid and nucleotide sequences were collected using an automated, iterative search method from *Entrez* Protein (GenPept) and *Entrez* Nucleotide (GenBank) (see Methods and Additional file 1: Table S1). Character matrices were generated in Mesquite 2.75 and aligned using Muscle 3.8.31) and the longest sequence for each species’ homologue(s) was retained [37,38]. Amino acid and nucleotide trees were generated under maximum parsimony using TNT 1.1 and Bayesian inference using MrBayes 3.2 [39,40].

**Figure 2:**
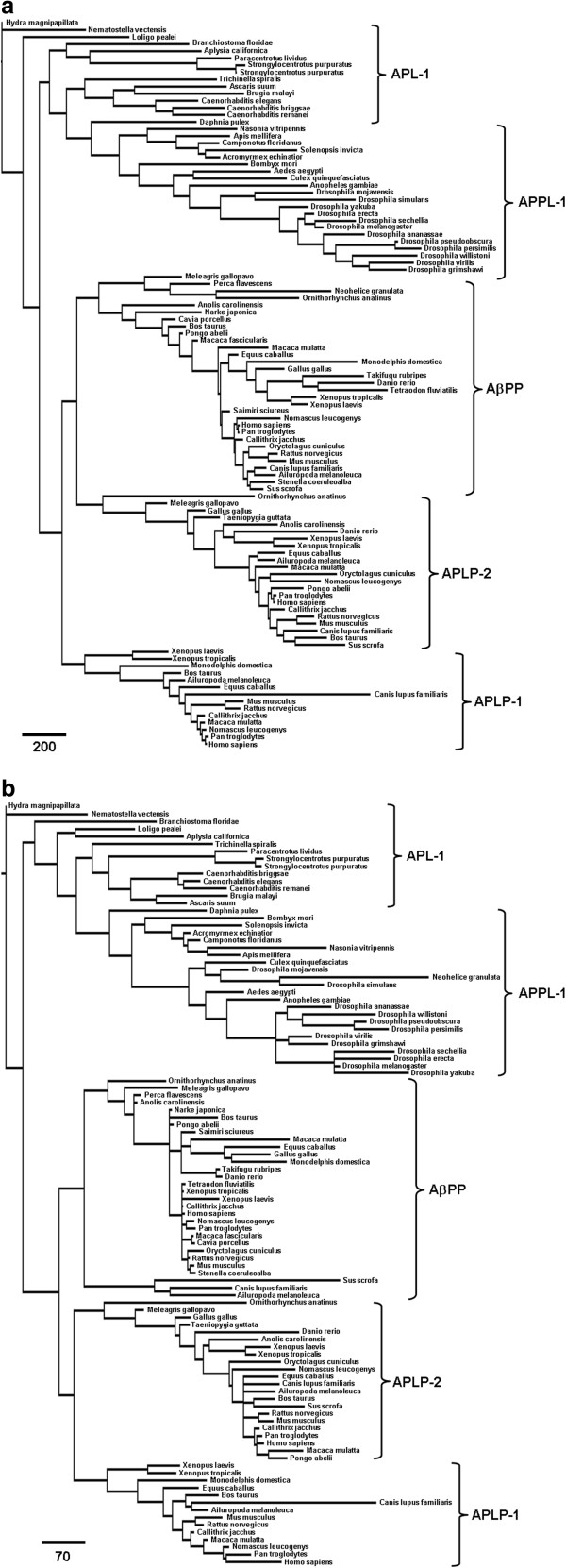
**Phylogenetic Relationships of 103 members of the Amyloid-β Precursor Protein Gene Family.** Shown are: (**a**) Phylogram showing the evolutionary relationships among the nucleotide sequences of the AβPP gene family; (**b**) Phylogram for the corresponding protein sequences. Trees were generated by maximum parsimony methods. Scale bars indicate character changes contributing to branch lengths.

The overall topology of the nucleotide and amino acid trees is similar among trees generated by maximum parsimony (Figure 2a and b) and Bayesian inference (Additional file 1: Figure S1a and b). Branch support data for the Bayesian analyses are found in Additional file 1: Figure S1 and support for the maximum parsimony analyses are found in Additional file 1: Figure S2.

The presence of an AβPP-like sequence in hydra (*Hydra magnipapillata*) and sea anemone (*Nematostella vectensis*) genomes suggests that the ancestral gene arose around metazoic divergence in the Ediacaran period, between 630–540 million years ago (Mya). No related sequences from single-celled organisms were found. A single member of the gene family has persisted across invertebrate species with a major divergence around the evolution of arthropods during the Cambrian period giving rise to APPL-1 (~500 Mya). Two gene duplication events occurred during the evolution of vertebrate species. Our search recovered a single AβPP-like gene for the cephalochordate lancelet (*Branchiostomidae floridae*) genome that was more closely related to mollusks and cnidarians than vertebrate sequences. The cartilaginous ray (*Narke japonica*) genome contains a single AβPP gene with high homology to human AβPP. The results indicate that AβPP and APLP-2 genes are present in the zebrafish (*Danio rerio*) but only AβPP was recovered for other members of class Osteichthyes (*Takifugu rubripes, Tetraodon fluviatilis, and Perca flavescens*). The majority of tetrapod genomes in this study contained all three members of the vertebrate AβPP gene family. Sequences for all three genes were found for Xenopus species, but APLP-1 sequences were not found for any members of class Aves or Reptilia.

Within the gene family, the nucleotide sequence phylogenetic trees (Figure 2a) indicate that AβPP and APLP-2 are more closely related than APLP-1 and APLP-2. Furthermore, the placement in the nucleotide phylogenetic tree suggests that APLP-1 may be the original vertebrate sequence. However, placement of the AβPP branch on the nucleotide tree is weakly supported under both maximum parsimony (47% resampling support, 12% relative Bremer; see Additional file 1: Figure S2a, b) and Bayesian inference (60% posterior probability; see Additional file 1: Figure S1a). In the amino acid sequence phylogenetic trees, APLP-1 and APLP-2 are more closely related and AβPP appears to be the original vertebrate peptide (Figure 2b). This arrangement has higher support for the placement of AβPP (65% resampling support, 100% relative Bremer support [maximum parsimony] Additional file 1: Figure S2c, d; 100% posterior probability [Bayesian inference]; Additional file 1: Figure S1b).

### Persistence of amyloid-β

The variability in the essential nature of the AβPP gene family can be observed by analyzing the evolutionary differences between related genes and shared residues according to specific functional domains. This was accomplished using synapomorphy frequency histograms. A synapomorphy is a trait or character shared by sister taxa of a clade that was derived from a previous common ancestor but not shared by taxa from another clade. Thus, synapomorphies contribute to the topology of a phylogenetic tree as factors in defining nodes on the tree [41]. Using the TNT program we collected synapomorphies present at each node of the consensus amino acid tree and examined the frequency of synapomorphy for each character across the sequence matrix. High frequencies of synapomorphy indicate residue changes at a given position make large contributions to the topology of the phylogenetic tree (conversely, low frequencies on the plots are associated with highly conserved domains/characters present in all terminal taxa groupings on the tree).

**Figure 3:**
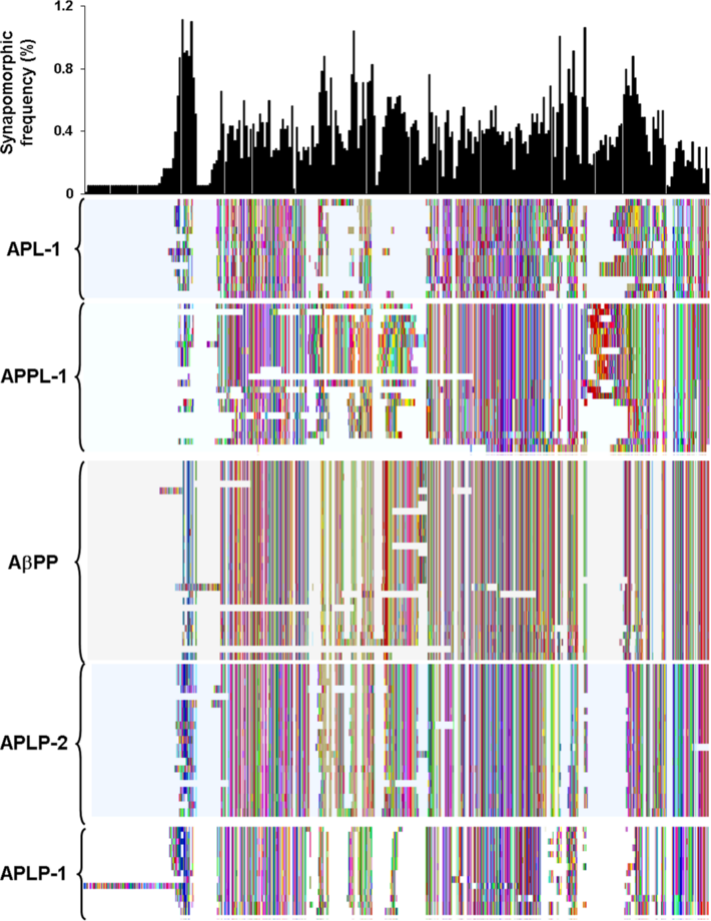
**Synapomorphic Character Frequencies for the Amyloid-β Precursor Protein Gene Family.** Histogram of synapomorphic frequency generated for the whole gene family above the aligned amino acid character map. The colors of the character map were arbitrarily assigned by Mesquite. Lack of a colored line indicates a gap in the aligned sequences. The five major branches of the AβPP phylogenetic tree are indicated to the left of the map. The histogram is binned at 5 residues and scaled as a percentage.

**Figure 4:**
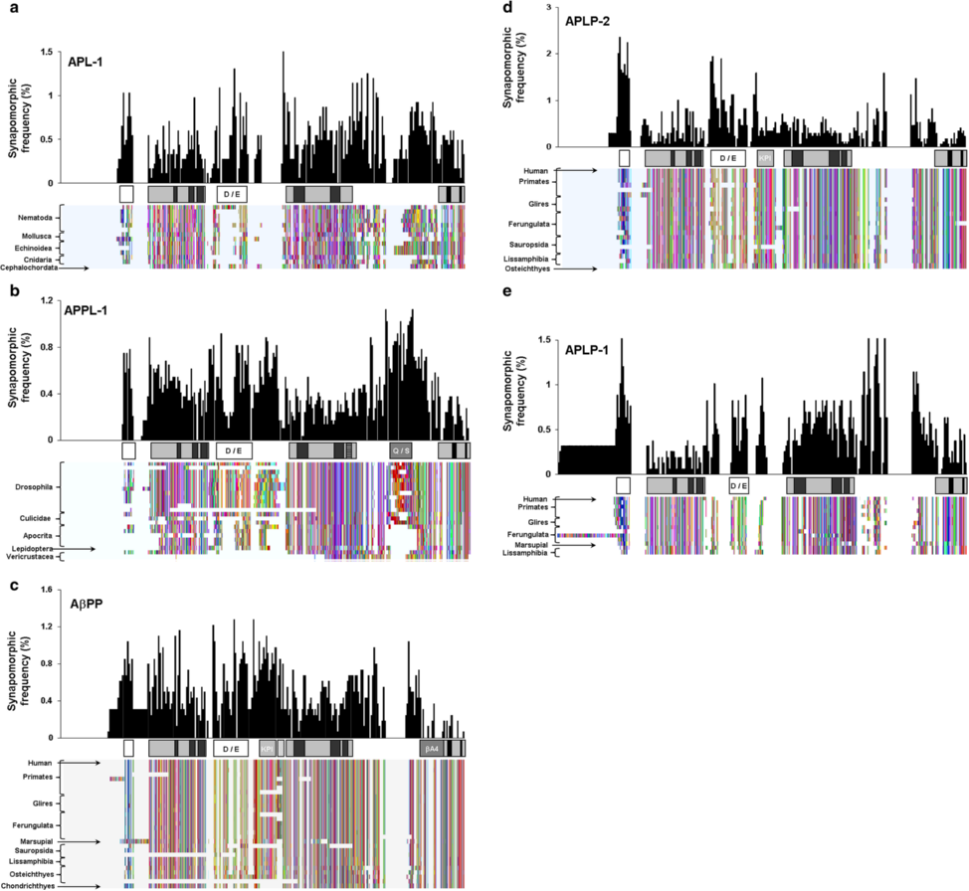
**Synapomorphic Frequencies Correspond to Conserved Sequences in the Amyloid-β Precursor Protein Gene Family.** Histograms of synapomorphic frequency generated for each major branch of the gene family above the representative schematic for each member and the amino acid character map. The colors of the character map were arbitrarily assigned by Mesquite. Lack of a colored line indicates a gap in the aligned sequences. Relevant taxonomic/cladistic classifications are indicated to the left of the maps. (**a**) APL-1; (**b**) APPL-1; (**c**) AbPP; (**d**) APLP-2; and (**e**) APLP-1 histograms are binned at 5 residues and scaled as percentages. Descriptions of the schematic regions are found in Figure 1.

In this study, synapomorphies were first analyzed across all positions in the dataset (Figure 3) and then analyses were stratified by the major branches corresponding to APPL-1, APL-1, AβPP, APLP-2, and APLP-1 (Figure 4, Tables 1 and 2). The most highly variable region was the E2 domain, which accounted for 18.3% of synapomorphies on the whole tree, while the most highly conserved domain was the E3 domain (which had 6.9% of the residue synapomorphies). The E3 region of AβPP, encoded by exons 17 and 18, contributed 1.7% of the synapomorphic frequencies to that branch and 0.3% for the whole tree. By contrast, the same region of the other homologues contributed between 1.2 – 2.4% for the whole tree and 6.3 – 12.5% for each major branch. The βA4 domain, encoded by exons 16 and 17, contributed 0.5% and 3.1% of synapomorphies to the whole tree and AβPP branch, respectively. The exon 16–17 regions of APLP-1 and APLP-2 contributed 1.5% and 1% of synapomorphies to the whole tree, respectively, and 8.8% and 5.4% to their respective branches. These data showed the E3 region to be the most highly conserved part of the entire gene family and conservation of the βA4/E3 region is even stronger for vertebrate species.

**Table 1:** Synapomorphic frequencies within conserved domains

**Domain**	**Whole tree**	**APL-1**	**APPL-1**	**AβPP**	**APLP-2**	**APLP-1**
N-Terminal Signal Peptide	11.61	1.25	1.49	2.05	3.31	3.57
E1	14.86	2.71	5.71	3.50	2.43	1.09
E2	18.33	4.95	4.18	3.22	2.83	4.17
E3	6.89	2.44	1.96	0.30	1.19	1.39

APL-1: amyloid precursor like 1 protein; APPL-1: AβPP-like 1 protein; AβPP: amyloid-β precursor protein; APLP-2: amyloid precursor protein like protein 2; APLP-1: amyloid precursor protein like protein 1. This table shows the percentage of synapomorphy accounted for by the N-terminal signal peptide and the three major conserved regions of the peptide sequences for the entire phyologenetic tree and the relative contribution for the five major branches.

**Table 2:** Synapomorphic frequencies of domains within each branch

**Domain**	**APL-1**	**APPL-1**	**AβPP**	**APLP-2**	**APLP-1**
N-Terminal Signal Peptide	6.41	4.80	11.74	18.47	21.38
E1	13.91	18.36	20.07	13.56	6.54
E2	25.38	13.42	18.49	15.81	24.94
E3	12.50	6.30	1.70	6.63	8.31

APL-1: amyloid precursor like 1 protein; APPL-1: AβPP-like 1 protein; AβPP: amyloid-β precursor protein; APLP-2: amyloid precursor protein like protein 2; APLP-1: amyloid precursor protein like protein 1. This table shows the percentage of synapomorphy accounted for by the N-terminal signal peptide and the three major conserved regions of the peptide sequences within each branch of the phylogenetic trees.

### Evolutionary relationships of amyloid-β formation potential

Deposition of Aβ has been well documented in mammals; the sequence is generally >95% identical across mammals and all vertebrates express β- and γ-secretases [42-44]. The Guinea pig rodent (*Cavia porcelus*) and the common hare (*Oryctolagus cuniculus*) have been shown to generate Aβ plaques, but neither the *Mus musculus* nor *Rattus norvegicus* rodents naturally produce Aβ plaques [45-48]. Evidence of Aβ accumulation in other vertebrate species is sparse. Deposition of extracellular Aβ has only been documented in one member of class Osteichthyes: *Onchyrus* sockeye salmon; the sockeye AβPP gene has not been sequenced [49]. While some species of birds may generate Aβ plaques or vascular amyloid deposition, there is no evidence of plaque formation or extracellular deposition in reptiles and amphibians despite >90% sequence homology [47,50]. No natural invertebrate amyloid-β plaques have been documented. Recently it was shown that the corresponding peptide from Drosophila can form an amyloid *in vivo* when co-expressed at high levels with the endogenous β-secretase gene [34].

**Figure 5:**
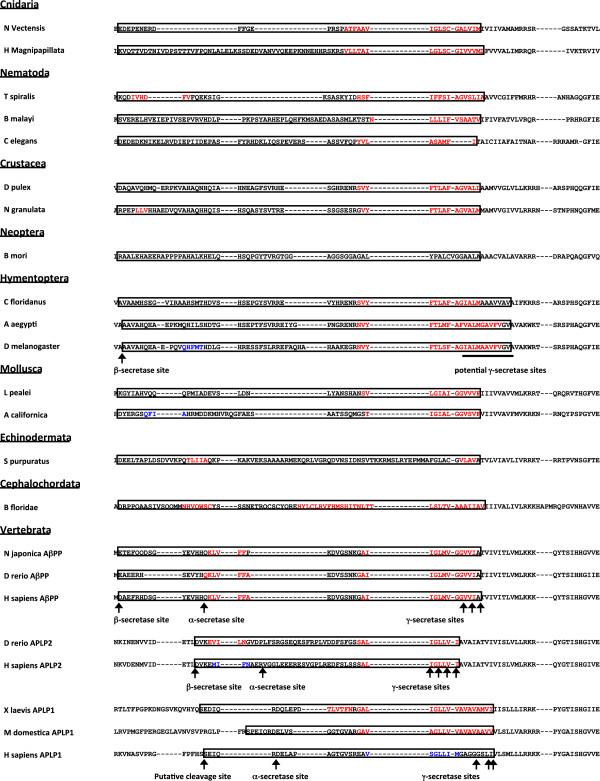
**Amyloidogenic Potential in the Amyloid-β Sequence.** Aligned representative amino acid sequences for the regions corresponding to exons 16 – 17 of human AβPP. Sequences tested are marked with boxes. Residues with AmylPred consensus and PASTA energies < − 4 are in red; residues with AmylPred consensus and PASTA energies between – 3 and – 4 are in blue. Known secretase cleavage sites are marked by arrows.

**Figure 6:**
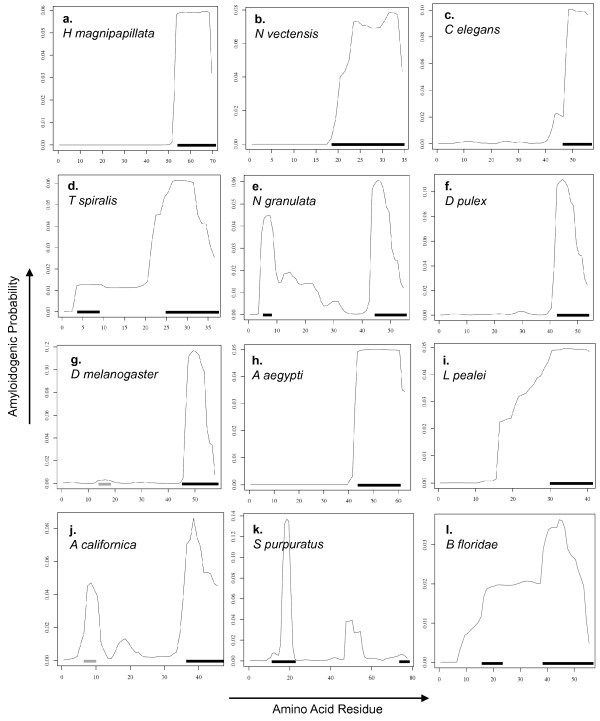
**Amyloid Potential in Invertebrate Amyloid-β Sequence.** Plots of probability of aggregation and stabilization of β-fibrils for each amino acid residue from PASTA for representative species. (**a**) *Hydra magnipapillata*, (**b**) *Nematostella vectensis*, (**c**) *Caenorhabditis elegans*, (**d**) *Trichinella spiralis*, (**e**) *Neohelice granulata*, (**f**) *Daphnia pulex*, (**g**) *Drosophila melanogaster*, (**h**) *Aedes aegypti*, (**i**) *Loligo pealei*, (**j**) *Aplysia californica*, (**k**) *Stronglyocentrotus pupuratus*, and (**l**) *Branchiostoma floridae*. Residues with PASTA energies < − 4 and AmylPred consensus are marked with a black line; residues with PASTA energies between – 3 and – 4 and AmylPred consensus are marked with a grey line.

**Figure 7:**
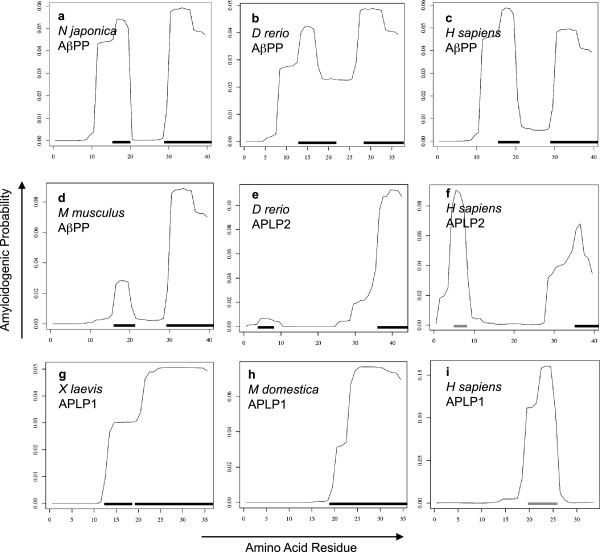
**Amyloid Potential in Vertebrate Amyloid-β Sequence.** Plots of probability of aggregation and stabilization of β-fibrils for each amino acid residue from PASTA for representative species. (**a**) *Narke japonica* AβPP, (**b**)* Danio rerio* AβPP, (**c**) *Homo sapiens* AβPP, (**d**) *Mus musculus* AβPP, (**e**) *Danio rerio* APLP2, (**f**) *Homo sapiens* APLP2, (**g**) *Xenopus laevis* APLP1, (**h**) *Monodelphis domestica* APLP1, and (**i**) *Homo sapiens* APLP1. Residues with PASTA energies < − 4 and AmylPred consensus are marked with a black line; residues with PASTA energies between – 3 and – 4 and AmylPred consensus are marked with a grey line.

In order to determine when Aβ formation first arose in evolution, we modeled β-sheet aggregation and amyloid formation probabilities for sequences corresponding to the human βA4 region using the AmylPred tool and PASTA server, both of which have been designed and validated using Aβ [51-54]. We found the C-terminal region of βA4 domains to have a high probability to form an amyloid or aggregate for nearly all sequences (Figures 5, 6, and 7). Only sequence from the silkmoth *Bombyx mori* had no amyloidogenic potential using both methods, although AmylPred predicted amyloidogenicity in a short C-terminal region. Very few sequences with amyloidogenic potential were found in the N-terminal region of invertebrate βA4 domains. The cnidarian sea anemone *Nematostella vectensis* and hydra *Hydra magnipapillata* exhibit strong C-terminal amyloid potential but little potential for amyloid formation in their N-terminal βA4 (Figure 6a and b). The nematode *Trichinella spiralis* is the only worm in our dataset with strongly amyloidogenic βA4 N-terminal sequences predicted by both methods (Figure 6d). The *Neohelice granulata* crab has a short N-terminal amyloid prone region (Figure 6e), but the water flea *Daphnia pulex* does not (Figure 6f). The Drosophila flies all express a potentially amyloidogenic N-terminal sequence predicted by AmylPred but with a PASTA energy of – 3.01 (Figure 6g), but no other members of Hymenoptera express amyloid prone sequence at the N-terminus. The squid *Loligo pealei* has PASTA energies < − 4 for long stretches of the N-terminal βA4 region but no consensus support from AmylPred (Figure 6i). The sea slug *Aplysia californica* has a short region with probable amyloid forming potential supported by AmylPred and with a PASTA energy of – 3.16 (Figure 6j). The sea urchins *Stronglyocentrotus purpuratus* and *Paracentrotus lividus* also had a short N-terminal region predicted to form amyloid (Figure 6k). The cephalochordate lancelet *Branchiostoma floridae* had two long N-terminal regions with high amyloidogenic potential (Figure 6l). All AβPP sequences in the dataset exhibited a strongly amyloidogenic N-terminal region, though the rodent *M musculus* and *R novergicus* sequences had reduced PASTA energies compared to other vertebrates for their N-terminal regions (Figures 5 and 7). Interestingly, *Danio rerio* APLP2 showed an N-terminal amyloidogenic region (Figure 7e) while all other APLP2 sequences were identical at these residues and had a lower probability of forming an amyloid (Figure 7f). The APLP1 sequences for *Xenopus laevis* and *Monodelphis domestica* showed long sections of aggregation prone sequence (Figure 7g and h). The remaining APLP1 sequences, representing only placental mammals, show a region with lowered probability of aggregation or fibril formation (Figure 7i).

## Discussion

This study provides the most comprehensive phylogeny of the AβPP gene family based on available data to date. The analysis reveals that the ancestral sequence evolved during metazoic divergence, which is much earlier than previously thought. The results further suggest that AβPP itself was the first vertebrate sequence and that APLP-1 and 2 are likely derived from gene duplication of AβPP.

It is possible that the vertebrate gene family arose as a duplication of APLP-1 followed by a second duplication to form APLP-2 and AβPP. However, it is also possible that the original duplication gave rise to APLP-2 and AβPP after which a duplication of APLP-2 gave rise to APLP-1. The search strategy used in this study found APLP-1 sequences only in tetrapods, AβPP in both cartilaginous and bony fish, and APLP-2 in one bony fish and most tetrapods.

**Figure 8:**
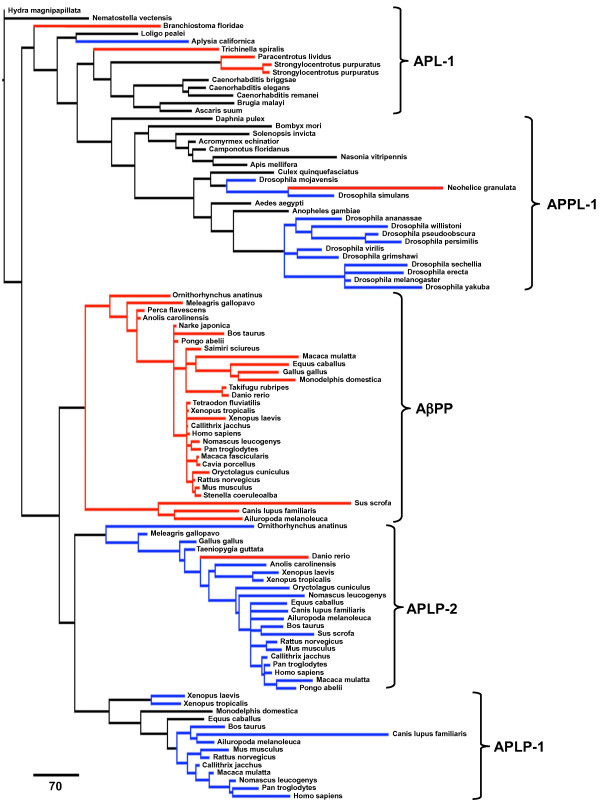
**Evolutionary Relationship of Amyloid-β Formation Potential.** Phylogram of the protein sequence for the AβPP family color-coded by prediction of Amyloid-β formation. Red: High potential (PASTA energies < − 4 and AmylPred consensus); Blue: Low potential (PASTA energies between – 3 and – 4 and AmylPred consensus); Black: No potential. Species with unique sequences demonstrating potential for Amyloid-β formation are labeled.

We found that the E3 C-terminal region of the protein is essentially unchanged since the divergence of jawed vertebrates during the Ordovician period and that amyloidogenic Aβ was present around this evolutionary step. Its persistence is likely due to the overlap with the E3 domain. It has been shown that the E3 domain is essential for life in mammals and the βA4 domain contains an HD motif with evidence of positive selection, both of which may explain some of the persistence of amyloidogenic Aβ in the mammalian genome [30,35]. Our analysis also found evidence of aggregation prone C-terminal regions in nearly all sequences in the dataset, which is not surprising as this is part of the transmembrane region high in hydrophobic residues, but a stable β-fold requires two regions within the peptide. Sequences with two separate domains capable of forming and stabilizing an amyloid were rare in protostomes, suggesting the characteristic developed after the divergence of deuterostomes and protostomes or was subsequently lost through mutation (Figure 8). Of particular note, the Drosophila sequence is predicted to form an amyloid but at a lowered probability than mammalian Aβ and there is experimental evidence that it can form fibrillar Aβ *in vivo*[34]. As no other Hymenoptera species in the study show amyloid potential, this likely represents a new mutation in the development of the fruitfly species. Interestingly, non-vertebrate deuterostome species in this study have amyloidogenic sequence but little homology to the mammalian Aβ sequence, suggesting that early amyloid prone regions may have evolved prior to the divergence of echinoderms, hemichordate, and chordate species. The main sequence variations arise from the N-terminal region aligned to *Homo sapiens* AβPP exon 16. APLP-2 from the zebrafish *Danio rerio* also showed amyloidogenic potential and all other APLP-2 had reduced potential to form Aβ. This is may be a result of mutation or indels in the exon 16 region during or after the gene duplication events giving rise to APLP-1 and APLP-2.

Because the data used in this study were based on *in silico* search strategies from deposited sequences in public repositories (GenBank and GenPept), it cannot be assumed that these data are necessarily complete for each species (i.e., a *de novo* sequencing was not performed for each species studied). Nonetheless, these data support the hypotheses that AβPP is the ancestral sequence for vertebrates, gene duplication after the speciation of cartilaginous and bony fish gave rise to APLP-2, and a subsequent partial or degenerate duplication of APLP-2 following the speciation of tetrapods gave rise to APLP-1. Some species may have subsequently lost either APLP-1 or APLP-2 genes.

The sequence difference in *Mus musculus* and *Rattus norvegicus* results from three amino acid substitutions from three single nucleotide changes. Whether the lack of amyloidogenesis in these particular rodents comes from these three changes or from other physiological considerations is unclear, but the presence of identical sequence in other rodents and mammals in general suggests that the ancestral species to mice and rats evolved around amyloidogenic Aβ. The lack of data on Aβ deposition in fish, birds, reptiles, and amphibians also suggests unknown physiological adaptations may limit Aβ production or deposition. Recently a mutation encoding a change from alanine to threonine at position 673 of AβPP was found to be protective against developing Alzheimer Disease, likely through reduction of β-secretase processing at that site [10]. It is interesting to note that all fish sequences in this study, with the exception of *Danio rerio*, have a threonine at this position, suggesting β-secretase processing may be reduced in these animals. In addition to processes that may increase or decrease Aβ production by regulating secretase efficiency or transcription, the presence of a β-secretase in the gene repertoire is an important consideration.

A whole genome assembly for *Nematostella vectensis* indicate the presence of the secretases but no studies have examined amyloid formation [55]. A genome for *Hydra magnipapillata* predicted the presence of a γ-secretase, but not a β-secretase (REFSEQ NW_002165109). Experimental evidence suggests that the nematode *Caenorhabditis elegans* does not express a β-secretase, although both α- and γ-secretases have been identified [56]. A search of Entrez Nucleotide returned no β-secretase sequences for other nematodes, crustaceans, hymenoptera, or lepidoptera in our dataset.

The increased understanding of disease genetics and increasing availability of molecular sequence data provide an opportunity to harness evolutionary approaches to provide deep insights pertaining to the etiology of disease. Using this approach we found the AβPP family to have origins in the speciation of the metazoic lineage and propose that ancestral Aβ may have arisen as deuterostomia and protostomia diverged. However, other mutations may continue to produce amyloidogenic sequences in this domain, as seen with Drosophila or unknown physiological factors may play a role in preventing Aβ formation as in mice and rats. The approach developed here may be widely applicable to the study of other critical disease genes and builds a foundation for further studies on the co-evolution of Alzheimer Disease associated proteins (e.g., co-evolution of ApoE or β-secretase with AβPP) that may yield novel approaches to treating or preventing Aβ formation.

## Methods

### Dataset collection and alignment

Amino acid sequences were collected through Entrez Protein using a combination of search terms and sequence similarity searches. First, based on previous studies of sequences from the Amyloid-β Precursor Protein [2-4,36] family five sets of metadata-based search terms developed and used to identify those sequences from across the Amyloid-β Precursor Protein family: (1) "App"[gene name] AND "animals"[porgn:__txid33208]; (2) "aplp1"[gene name] and "animals"[porgn:__txid33208]; (3) "aplp2"[gene name] and "animals"[porgn:__txid33208]; (4) "apl-1"[gene name] and "nematodes"[porgn:__txid6231]; and (5) "app_amyloid". Sequences for which the organism was either “Unknown” or listed as a “synthetic construct” were removed. Next, a stringent (E-value = 0.0) blastp (BLAST+ v.2.2.26) was used to search Entrez Protein for potential orthologous amino acid sequences for each of the sequences identified in the metadata-based search from the non-redundant protein database. An additional stringent blastp search was then done iteratively for each new sequence identified, until no additional sequences were found. The resulting dataset (which contained 435 sequences) was then subjected to multiple sequence alignment using MUSCLE v.3.8.31 [38]. The multiple sequence alignment was manually inspected (by viewing the data in Mesquite 2.75 [37]) to identify the one longest representative sequence per taxon (e.g., only the sequence for human AβPP770 which contains all transcribed and translated exons was kept). As sequences were removed from the dataset, the multiple sequence alignment was redone. The resulting dataset reflected 103 taxa corresponding to 67 species. Based on identifiers within GenPept records, corresponding nucleic acid sequences were then collected for each amino acid sequence. These nucleotide sequences were also subjected to multiple sequence alignment using MUSCLE. Character maps were generated using the Mesquite character matrices.

### Generation of phylogenetic trees

Trees were obtained by maximum parsimony using TNT 1.1 and Bayesian inference using MrBayes 3.2.0 [39,40]. For analyses in TNT, the ‘aquickie.run’ script was used to guide the search, which aimed to find the optimal score 20 times independently, using defaults of "xmult" plus 10 cycles of tree-drifting. This resulted in 131 nucleotide trees from more than 8x10^8^ rearrangements and 103714 amino acid trees from more than 7x10^8^ rearrangements. For consensus tree calculation, trees were TBR-collapsed, Bremer group supports calculated by TBR-swapping, and bootstrap resampling by 100 replications of symmetric resampling with a single random addition (see Additional file 1).

For MrBayes, the Metropolis-coupled Markov chain Monte Carlo analysis was set for 2 runs with 4 chains each with a temperature of 0.2 degrees. A General Time Reversible (GTR) model with a Dirichlet (flat) probability distribution of nucleotide rate change parameters, stationary nucleotide frequencies, no specified shape parameter for the gamma distribution of rate variation, and no invariable sites was used for the nucleotide analyses; this is the default prior model for nucleotide matrices in MrBayes. All runs favored the WAG rate matrix as the prior model for the amino acid analyses [57].

Markov Chain analysis was continued until the runs converged, when the standard deviation of the split frequencies remained <0.01 and likelihood analysis found the potential scale reduction factor approached 1.0 [58]. For the nucleotide modeling this took more than 3x10^6^ generations; for the protein analysis this took more than 2x10^6^ generations. Consensus trees were constructed using the 50% majority rule with 95% cumulative posterior probability from 925 nucleotide trees and 1,591 amino acid trees (see Additional file 1). All tree diagrams were generated in either Dendroscope 3.1.0 or FigTree 1.3.1 [59,60].

### Synapomorphic frequencies

Unambiguous synapomorphies at each node were generated in TNT for the maximum parsimony consensus trees. The frequency of a given character being synapomorphic at a given node was examined for the entire amino acid tree and for each of the five major branches. Probabilistic models of synapomorphy have been developed to address the confounding of homoplasy and lend statistical support to defining a character as synapomorphic as opposed to homoplasious [41]. While these are important considerations for higher resolution analysis of a gene family, use of simple statistical analysis for such a large and diverse dataset is a reasonable approach to defining areas of conservation or change, accepting internal error for random mutation producing homoplasy or loss of an actual synapomorphy.

### Aggregation modeling

There are a number of programs available for modeling β-folding and aggregation of amyloidogenic peptides [61]. AmylPred is a consensus tool that predicts β-folding and aggregation based on a set of five published methods and uses agreement of 2 or more methods for determining consensus [54]. PASTA predicts stabilizing sequences in β-fibrillar structures using a calculation of the change of energy from pairing between amino acid sequences [53]. Regions that are known to form ordered β-fibril structures have a PASTA energy less than – 4. Using aligned amino acid sequences coded by *Homo sapiens* AβPP exons 16 and 17, we examined the corresponding βA4 region across all taxa and used known secretase cleavage sites to determine the aligned sequences for submission to AmylPred and PASTA [62-64]. Where cleavage sites are not known from previous studies, boundaries were chosen based on similar species and sequences. In cases where there was no clear similarity, boundaries were extended to correspond with *Homo sapiens* Aβ42. PASTA energies were collected until greater than – 2 by sequential truncation of the C-terminus for each sequence.

## Abbreviations

Aβ: Amyloid-β; AβPP: APP, Amyloid-β Precursor Protein; APPL-1: Amyloid-β Precursor Protein-like 1 protein; APLP-1: Amyloid precursor like protein 1; APLP-2: Amyloid precursor like protein 2; APL-1: Amyloid precursor like 1 protein; BLS: Basolateral sorting signal; GFLD: Growth-factor-like domain; KPI: Kunitz-protease inhibitor; Mya: Million years ago.

## Competing interests

INS and WGT do not have any conflicts of interest to disclose.

## Authors’ contributions

INS and WGT conceived of and designed the study together. INS collected and aligned the sequences. WGT conducted the tree building and aggregation analyses. Both INS and WGT interpreted the results and drafted the manuscript together. Both authors read and approved the final manuscript.

## Supplementary Material

Additional file 1 **Figure S1.** Phylogenetic Relationships among the Amyloid-β Precursor Protein Gene Family from Baysian Inference. **a**, Phylogram showing the evolutionary relationships among the nucleotide sequences of the AβPP gene family. **b**, Phylogram for the corresponding protein sequences. Trees were generated by Bayesian inference methods and show posterior probability values are each node. **Figure S2.** Branch Supports for Phylogenetic Trees. Symmetric bootstrap re-sampling and Bremer supports, for nucleotide trees (**a** and **b,** respectively) and for amino acid trees (**c** and **d**, respectively). **Table S1.** Taxa Species Names and Sequence Accession Numbers. Click here for file

## References

[B1] Hardy J Selkoe DJ The amyloid hypothesis of Alzheimer's disease: progress and problems on the road to therapeutics Science 2002 297 5580 353 356 10.1126/science.1072994 12130773

[B2] Guo Q Wang Z Li H Wiese M Zheng H APP physiological and pathophysiological functions: insights from animal models Cell Res 2012 22 1 78 89 10.1038/cr.2011.116 21769132 PMC3351924

[B3] Jacobsen KT Iverfeldt K Amyloid precursor protein and its homologues: a family of proteolysis-dependent receptors Cell Mol Life Sci 2009 66 14 2299 2318 10.1007/s00018-009-0020-8 19333550 PMC11115575

[B4] Walsh DM Minogue AM Sala Frigerio C Fadeeva JV Wasco W Selkoe DJ The APP family of proteins: similarities and differences Biochem Soc Trans 2007 35 Pt 2 416 420 17371289 10.1042/BST0350416

[B5] Greenwald J Riek R Biology of amyloid: structure, function, and regulation Structure 2010 18 10 1244 1260 10.1016/j.str.2010.08.009 20947013

[B6] Chernoff YO Amyloidogenic domains, prions and structural inheritance: rudiments of early life or recent acquisition? Curr Opin Chem Biol 2004 8 6 665 671 10.1016/j.cbpa.2004.09.002 15556413

[B7] Brack A Orgel LE Beta structures of alternating polypeptides and their possible prebiotic significance Nature 1975 256 5516 383 387 10.1038/256383a0 238134

[B8] Inge-Vechtomov SG Zhouravleva GA Chernoff YO Biological roles of prion domains Prion 2007 1 4 228 235 10.4161/pri.1.4.5059 19172114 PMC2634536

[B9] Rosenberg RN The molecular and genetic basis of AD: the end of the beginning: the 2000 Wartenberg lecture Neurology 2000 54 11 2045 2054 10.1212/WNL.54.11.2045 10851361

[B10] Jonsson T Atwal JK Steinberg S Snaedal J Jonsson PV Bjornsson S Stefansson H Sulem P Gudbjartsson D Maloney J A mutation in APP protects against Alzheimer's disease and age-related cognitive decline Nature 2012 advance online publication 10.1038/nature11283 22801501

[B11] Barrachina M Dalfo E Puig B Vidal N Freixes M Castano E Ferrer I Amyloid-beta deposition in the cerebral cortex in Dementia with Lewy bodies is accompanied by a relative increase in AbetaPP mRNA isoforms containing the Kunitz protease inhibitor Neurochem Int 2005 46 3 253 260 10.1016/j.neuint.2004.08.006 15670642

[B12] Yamada M Naiki H Cerebral amyloid angiopathy Prog Mol Biol Transl Sci 2012 107 41 78 22482447 10.1016/B978-0-12-385883-2.00006-0

[B13] Liang WS Dunckley T Beach TG Grover A Mastroeni D Ramsey K Caselli RJ Kukull WA McKeel D Morris JC Neuronal gene expression in non-demented individuals with intermediate Alzheimer's Disease neuropathology Neurobiol Aging 2010 31 4 549 566 10.1016/j.neurobiolaging.2008.05.013 18572275 PMC2844804

[B14] Kotzbauer PT Cairns NJ Campbell MC Willis AW Racette BA Tabbal SD Perlmutter JS Pathologic Accumulation of alpha-Synuclein and Abeta in Parkinson Disease Patients With Dementia Arch Neurol 2012 1 6 10.1001/archneurol.2012.1608 PMC3616136 22825369

[B15] Nikolaev A McLaughlin T O'Leary DD Tessier-Lavigne M APP binds DR6 to trigger axon pruning and neuron death via distinct caspases Nature 2009 457 7232 981 989 10.1038/nature07767 19225519 PMC2677572

[B16] Sinha S Lieberburg I Cellular mechanisms of beta-amyloid production and secretion Proc Natl Acad Sci U S A 1999 96 20 11049 11053 10.1073/pnas.96.20.11049 10500121 PMC34239

[B17] Thinakaran G Koo EH APP trafficking, processing and function J Biol Chem 2008 283 44 29615 10.1074/jbc.R800019200 18650430 PMC2573065

[B18] Xu F Davis J Miao J Previti ML Romanov G Ziegler K Van Nostrand WE Protease nexin-2/amyloid beta-protein precursor limits cerebral thrombosis Proc Natl Acad Sci U S A 2005 102 50 18135 18140 10.1073/pnas.0507798102 16330760 PMC1312400

[B19] Bush AI Martins RN Rumble B Moir R Fuller S Milward E Currie J Ames D Weidemann A Fischer P The amyloid precursor protein of Alzheimer's disease is released by human platelets J Biol Chem 1990 265 26 15977 15983 2118534

[B20] Joachim CL Mori H Selkoe DJ Amyloid beta-protein deposition in tissues other than brain in Alzheimer's disease Nature 1989 341 6239 226 230 10.1038/341226a0 2528696

[B21] Lee YH Tharp WG Maple RL Nair S Permana PA Pratley RE Amyloid precursor protein expression is upregulated in adipocytes in obesity Obesity (Silver Spring, Md) 2008 16 7 1493 1500 10.1038/oby.2008.267 18483477

[B22] Galloway S Jian L Johnsen R Chew S Mamo JC Deta-amyloid or its precursor protein is found in epithelial cells of the small intestine and is stimulated by high-fat feeding J Nutr Biochem 2007 18 4 279 284 10.1016/j.jnutbio.2006.07.003 16962759

[B23] Hansel DE Rahman A Wehner S Herzog V Yeo CJ Maitra A Increased expression and processing of the Alzheimer amyloid precursor protein in pancreatic cancer may influence cellular proliferation Cancer Res 2003 63 21 7032 7037 14612490

[B24] Herzog V Kirfel G Siemes C Schmitz A Biological roles of APP in the epidermis Eur J Cell Biol 2004 83 11–12 613 624 15679106 10.1078/0171-9335-00401

[B25] Kuo YM Kokjohn TA Watson MD Woods AS Cotter RJ Sue LI Kalback WM Emmerling MR Beach TG Roher AE Elevated abeta42 in skeletal muscle of Alzheimer disease patients suggests peripheral alterations of AbetaPP metabolism Am J Pathol 2000 156 3 797 805 10.1016/S0002-9440(10)64947-4 10702395 PMC1876838

[B26] Kang J Lemaire HG Unterbeck A Salbaum JM Masters CL Grzeschik KH Multhaup G Beyreuther K Muller-Hill B The precursor of Alzheimer's disease amyloid A4 protein resembles a cell-surface receptor Nature 1987 325 6106 733 736 10.1038/325733a0 2881207

[B27] Hornsten A Lieberthal J Fadia S Malins R Ha L Xu X Daigle I Markowitz M O'Connor G Plasterk R APL-1, a Caenorhabditis elegans protein related to the human beta-amyloid precursor protein, is essential for viability Proc Natl Acad Sci U S A 2007 104 6 1971 1976 10.1073/pnas.0603997104 17267616 PMC1794273

[B28] von Koch CS Zheng H Chen H Trumbauer M Thinakaran G van der Ploeg LH Price DL Sisodia SS Generation of APLP2 KO mice and early postnatal lethality in APLP2/APP double KO mice Neurobiol Aging 1997 18 6 661 669 10.1016/S0197-4580(97)00151-6 9461064

[B29] Heber S Herms J Gajic V Hainfellner J Aguzzi A Rulicke T von Kretzschmar H von Koch C Sisodia S Tremml P Mice with combined gene knock-outs reveal essential and partially redundant functions of amyloid precursor protein family members J Neurosci 2000 20 21 7951 7963 11050115 10.1523/JNEUROSCI.20-21-07951.2000 PMC6772747

[B30] Li H Wang Z Wang B Guo Q Dolios G Tabuchi K Hammer RE Sudhof TC Wang R Zheng H Genetic dissection of the amyloid precursor protein in developmental function and amyloid pathogenesis J Biol Chem 2010 285 40 30598 30605 10.1074/jbc.M110.137729 20693289 PMC2945554

[B31] Poeck B Strauss R Kretzschmar D Analysis of amyloid precursor protein function in Drosophila melanogaster Exp Brain Res 2012 217 3–4 413 421 21912928 10.1007/s00221-011-2860-3

[B32] Song P Pimplikar SW Knockdown of amyloid precursor protein in zebrafish causes defects in motor axon outgrowth PLoS One 2012 7 4 e34209 10.1371/journal.pone.0034209 22545081 PMC3335837

[B33] Joshi P Liang JO DiMonte K Sullivan J Pimplikar SW Amyloid precursor protein is required for convergent-extension movements during Zebrafish development Dev Biol 2009 335 1 1 11 10.1016/j.ydbio.2009.07.041 19664615

[B34] Carmine-Simmen K Proctor T Tschape J Poeck B Triphan T Strauss R Kretzschmar D Neurotoxic effects induced by the Drosophila amyloid-beta peptide suggest a conserved toxic function Neurobiol Dis 2009 33 2 274 281 10.1016/j.nbd.2008.10.014 19049874 PMC4418460

[B35] Miklos I Zadori Z Positive evolutionary selection of an HD motif on Alzheimer precursor protein orthologues suggests a functional role PLoS Comput Biol 2012 8 2 e1002356 10.1371/journal.pcbi.1002356 22319430 PMC3271017

[B36] Coulson EJ Paliga K Beyreuther K Masters CL What the evolution of the amyloid protein precursor supergene family tells us about its function Neurochem Int 2000 36 3 175 184 10.1016/S0197-0186(99)00125-4 10676850

[B37] Maddison WP Maddison DP Mesquite: a modular system for evolutionary analysis 2011 275

[B38] Edgar RC MUSCLE: multiple sequence alignment with high accuracy and high throughput Nucleic Acids Res 2004 32 5 1792 1797 10.1093/nar/gkh340 15034147 PMC390337

[B39] Goloboff P Farris J Nixon K TNT, a free program for phylogenetic analysis Cladistics 2008 24 774 786 10.1111/j.1096-0031.2008.00217.x

[B40] Ronquist F Huelsenbeck JP MrBayes 3: Bayesian phylogenetic inference under mixed models Bioinformatics 2003 19 12 1572 1574 10.1093/bioinformatics/btg180 12912839

[B41] Turjak M Trontelj P A method for measuring support for synapomorphy using character state distributions on phylogenetic trees Cladistics 2012 1 1 12 10.1111/j.1096-0031.2012.00403.x 34844375

[B42] Finch CE Sapolsky RM The evolution of Alzheimer disease, the reproductive schedule, and apoE isoforms Neurobiol Aging 1999 20 4 407 428 10.1016/S0197-4580(99)00053-6 10604433

[B43] Sarasa M Gallego C Alzheimer-Like Neurodegeneration as a Probable Cause of Cetacean Stranding Poster session presented at: 5th FENS Forum: 2006; Vienna, Austria 2006

[B44] Selkoe DJ Bell DS Podlisny MB Price DL Cork LC Conservation of brain amyloid proteins in aged mammals and humans with Alzheimer's disease Science 1987 235 4791 873 877 10.1126/science.3544219 3544219

[B45] Beach TG Physiologic origins of age-related beta-amyloid deposition Neurodegener Dis 2008 5 3–4 143 145 18322373 10.1159/000113685

[B46] Ionov ID Pushinskaya II Amyloid-beta production in aged guinea pigs: atropine-induced enhancement is reversed by naloxone Neurosci Lett 2010 480 1 83 86 10.1016/j.neulet.2010.06.010 20540990

[B47] Dayan AD Comparative neuropathology of ageing. Studies on the brains of 47 species of vertebrates Brain 1971 94 1 31 42 10.1093/brain/94.1.31 4324032

[B48] Flood DG Lin YG Lang DM Trusko SP Hirsch JD Savage MJ Scott RW Howland DS A transgenic rat model of Alzheimer's disease with extracellular Abeta deposition Neurobiol Aging 2009 30 7 1078 1090 10.1016/j.neurobiolaging.2007.10.006 18053619

[B49] Maldonado TA Jones RE Norris DO Timing of neurodegeneration and beta-amyloid (Abeta) peptide deposition in the brain of aging kokanee salmon J Neurobiol 2002 53 1 21 35 10.1002/neu.10090 12360580

[B50] Nakayama H Katayama K Ikawa A Miyawaki K Shinozuka J Uetsuka K Nakamura S Kimura N Yoshikawa Y Doi K Cerebral amyloid angiopathy in an aged great spotted woodpecker (Picoides major) Neurobiol Aging 1999 20 1 53 56 10.1016/S0197-4580(99)00004-4 10466893

[B51] Trovato A Seno F Tosatto SC The PASTA server for protein aggregation prediction Protein Eng Des Sel 2007 20 10 521 523 10.1093/protein/gzm042 17720750

[B52] Fernandez-Escamilla AM Rousseau F Schymkowitz J Serrano L Prediction of sequence-dependent and mutational effects on the aggregation of peptides and proteins Nat Biotechnol 2004 22 10 1302 1306 10.1038/nbt1012 15361882

[B53] Trovato A Chiti F Maritan A Seno F Insight into the structure of amyloid fibrils from the analysis of globular proteins PLoS Comput Biol 2006 2 12 e170 10.1371/journal.pcbi.0020170 17173479 PMC1698942

[B54] Frousios KK Iconomidou VA Karletidi CM Hamodrakas SJ Amyloidogenic determinants are usually not buried BMC Struct Biol 2009 9 44 10.1186/1472-6807-9-44 19589171 PMC2714319

[B55] Putnam NH Srivastava M Hellsten U Dirks B Chapman J Salamov A Terry A Shapiro H Lindquist E Kapitonov VV Sea anemone genome reveals ancestral eumetazoan gene repertoire and genomic organization Science 2007 317 5834 86 94 10.1126/science.1139158 17615350

[B56] Link CD C. elegans models of age-associated neurodegenerative diseases: lessons from transgenic worm models of Alzheimer's disease Exp Gerontol 2006 41 10 1007 1013 10.1016/j.exger.2006.06.059 16930903

[B57] Whelan S Goldman N A general empirical model of protein evolution derived from multiple protein families using a maximum-likelihood approach Mol Biol Evol 2001 18 5 691 699 10.1093/oxfordjournals.molbev.a003851 11319253

[B58] Gelman A Rubin D Inference from Iterative Simulation using Multiple Sequences Stat Sci 1992 7 457 511 10.1214/ss/1177011136

[B59] Huson DH Richter DC Rausch C Dezulian T Franz M Rupp R Dendroscope: An interactive viewer for large phylogenetic trees BMC Bioinformatics 2007 8 460 10.1186/1471-2105-8-460 18034891 PMC2216043

[B60] Rambaut A FigTree 2009 131

[B61] Hamodrakas SJ Protein aggregation and amyloid fibril formation prediction software from primary sequence: towards controlling the formation of bacterial inclusion bodies FEBS J 2011 278 14 2428 2435 10.1111/j.1742-4658.2011.08164.x 21569208

[B62] Hogl S Kuhn PH Colombo A Lichtenthaler SF Determination of the proteolytic cleavage sites of the amyloid precursor-like protein 2 by the proteases ADAM10, BACE1 and gamma-secretase PLoS One 2011 6 6 e21337 10.1371/journal.pone.0021337 21695060 PMC3117885

[B63] Yanagida K Okochi M Tagami S Nakayama T Kodama TS Nishitomi K Jiang J Mori K Tatsumi S Arai T The 28-amino acid form of an APLP1-derived Abeta-like peptide is a surrogate marker for Abeta42 production in the central nervous system EMBO Mol Med 2009 1 4 223 235 10.1002/emmm.200900026 20049724 PMC3378133

[B64] Minogue AM Stubbs AK Frigerio CS Boland B Fadeeva JV Tang J Selkoe DJ Walsh DM Gamma-secretase processing of APLP1 leads to the production of a p3-like peptide that does not aggregate and is not toxic to neurons Brain Res 2009 1262 89 99 19401174 10.1016/j.brainres.2009.01.008

