# Supplementary material for: Origins of amyloid-β
Source: BMC Genomics. 2013 Apr 30;14:290. doi: 10.1186/1471-2164-14-290 (PMC3660159; doi:10.1186/1471-2164-14-290)
Supplement: Additional file 1 — Figure S1. Phylogenetic Relationships among the Amyloid-β Precursor Protein Gene Family from Baysian Inference. a, Phylogram showing the evolutionary relationships among the nucleotide sequences of the AβPP gene family. b, Phylogram for the corresponding protein sequences. Trees were generated by Bayesian inference methods and show posterior probability values are each node. Figure S2. Branch Supports for Phylogenetic Trees. Symmetric bootstrap re-sampling and Bremer supports, for nucleotide trees (a and b, respectively) and for amino acid trees (c and d, respectively). Table S1. Taxa Species Names and Sequence Accession Numbers. [file 1471-2164-14-290-S1.pdf]

Tharp and Sarkar

Origins of Amyloid-β

Supplementary Data

Supplementary Figure 1 – Phylogenetic Relationships among the Amyloid-β Precursor Protein Gene Family from Baysian Inference. **a**, Phylogram showing the evolutionary relationships among the nucleotide sequences of the AβPP gene family. **b**, Phylogram for the corresponding protein sequences. Trees were generated by Bayesian inference methods and show posterior probability values are each node.

Supplementary Figure 2 – Branch Supports for Phylogenetic Trees. Symmetric bootstrap re-sampling and Bremer supports, for nucleotide trees (**a** and **b,** respectively) and for amino acid trees (**c** and **d**, respectively).

Supplementary Table 1 – Taxa Species Names and Sequence Accession Numbers

Supplementary Figure 1a

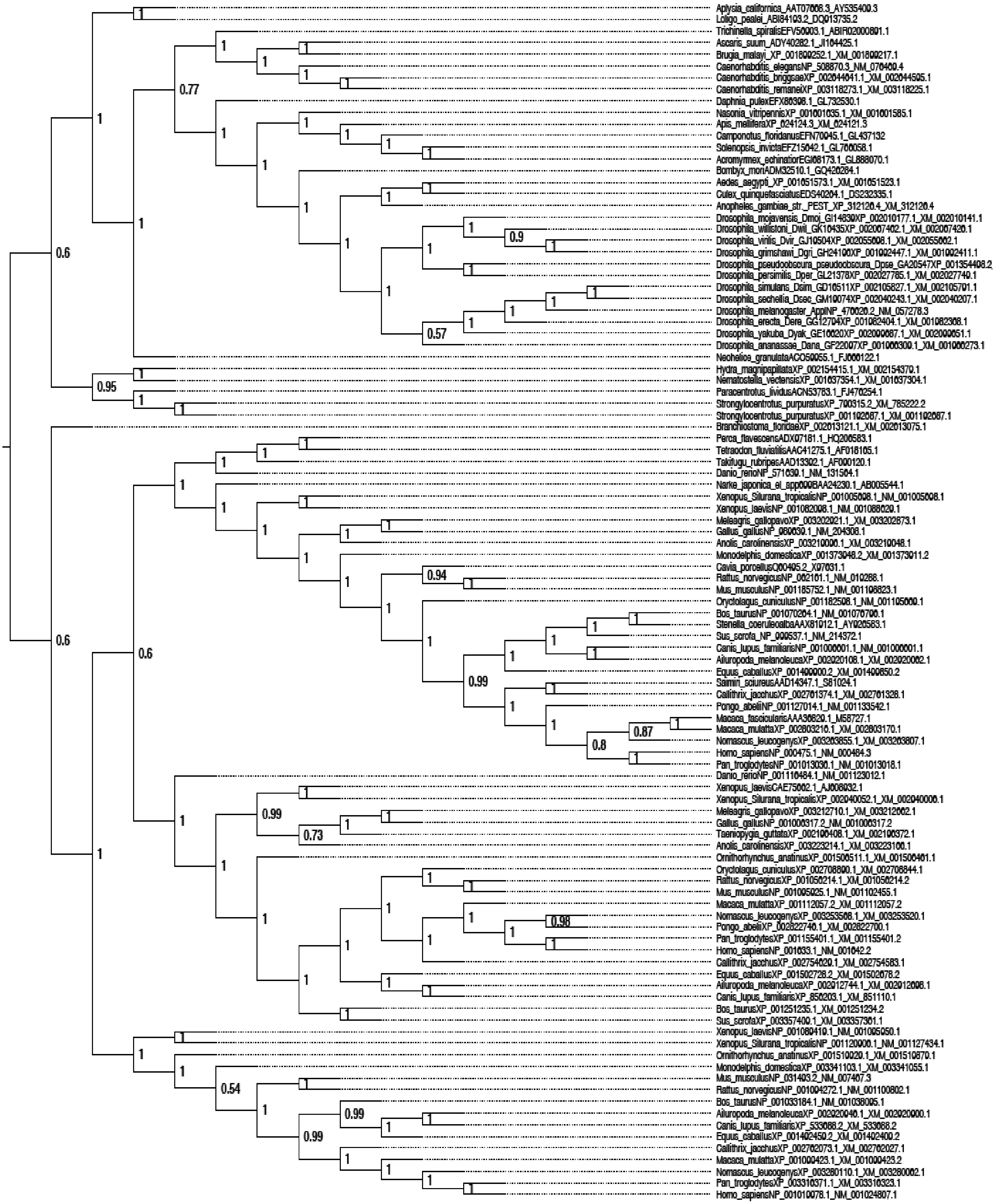

Supplementary Figure 1b

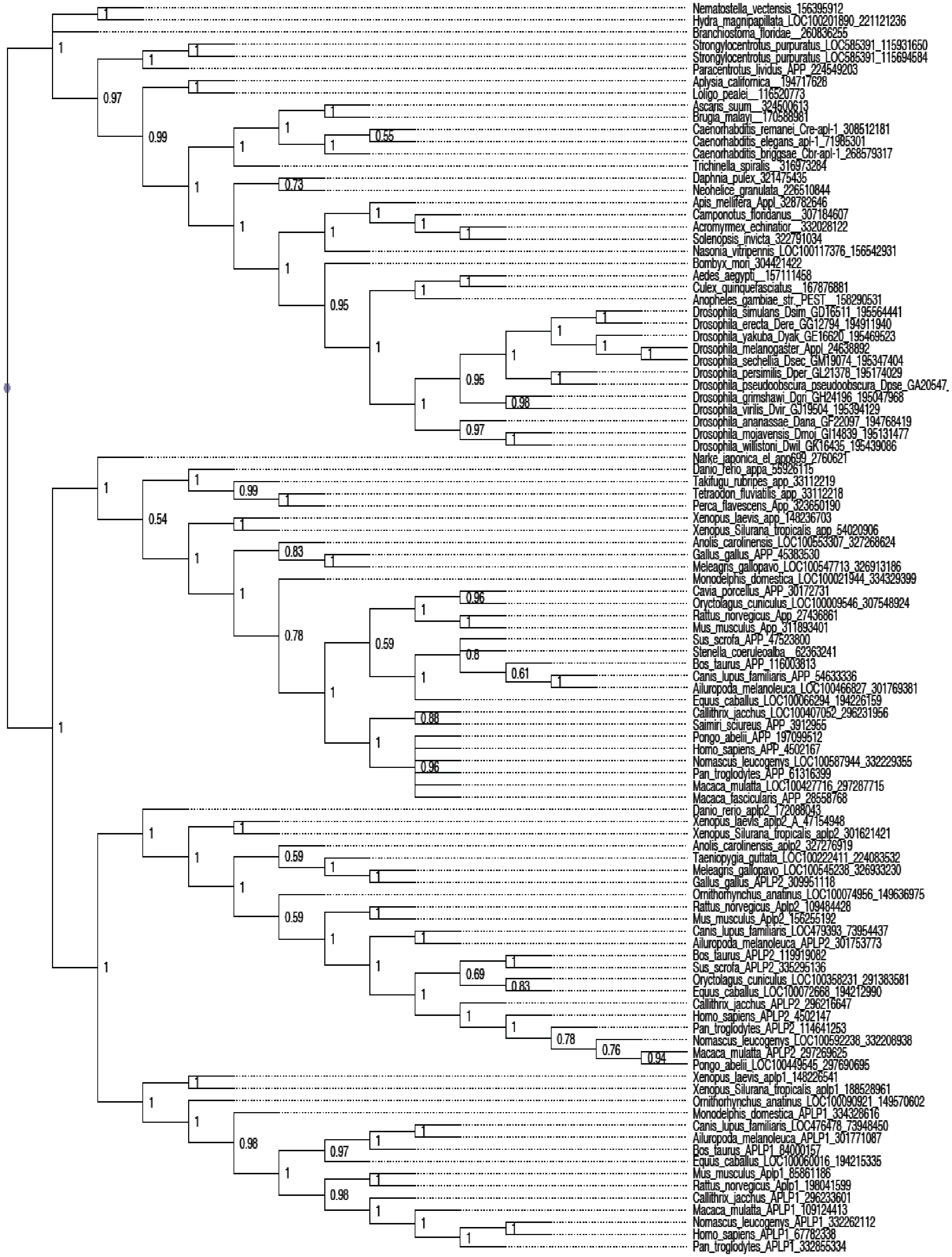

Supplementary Figure 2a

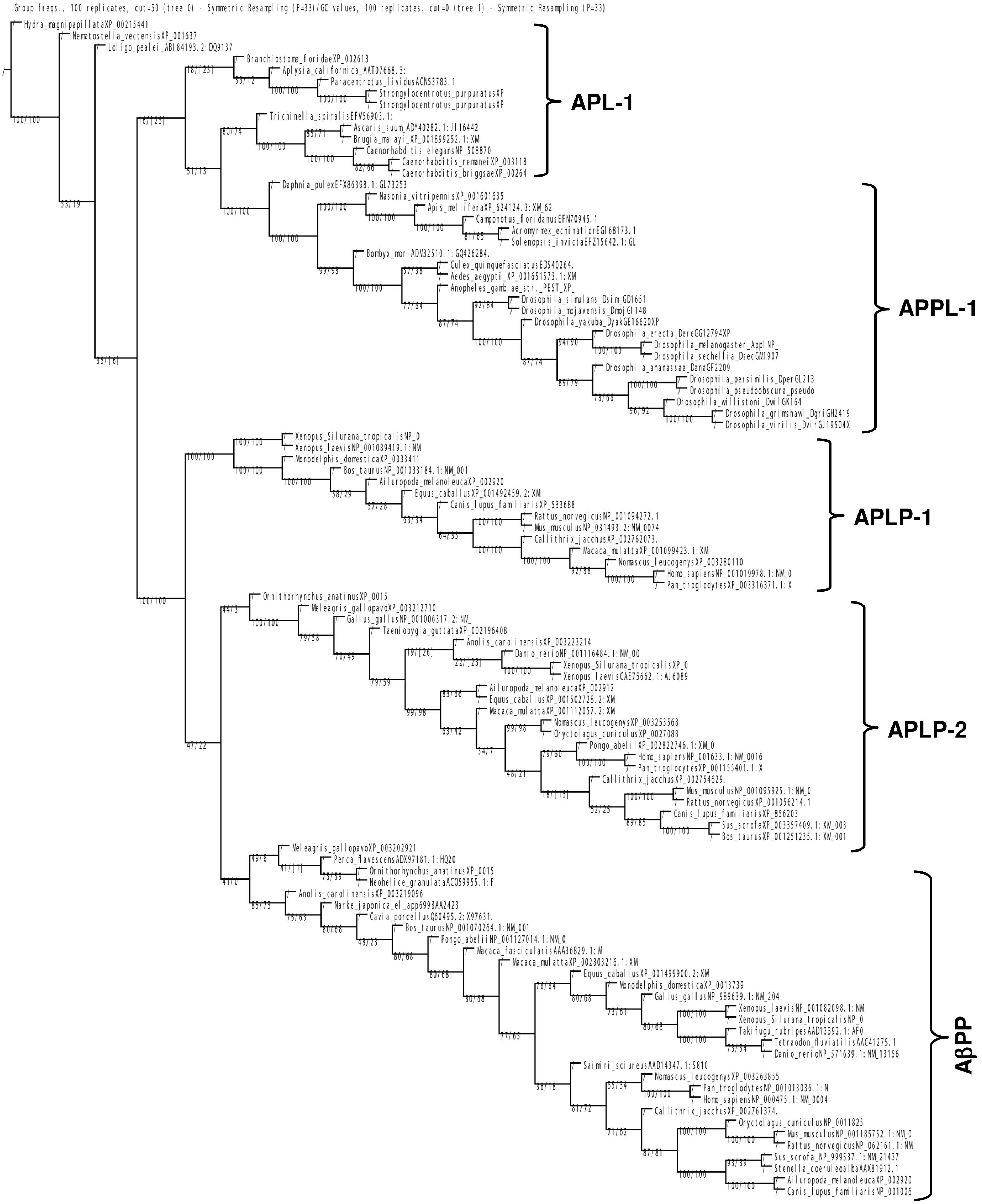

Supplementary Figure 2b

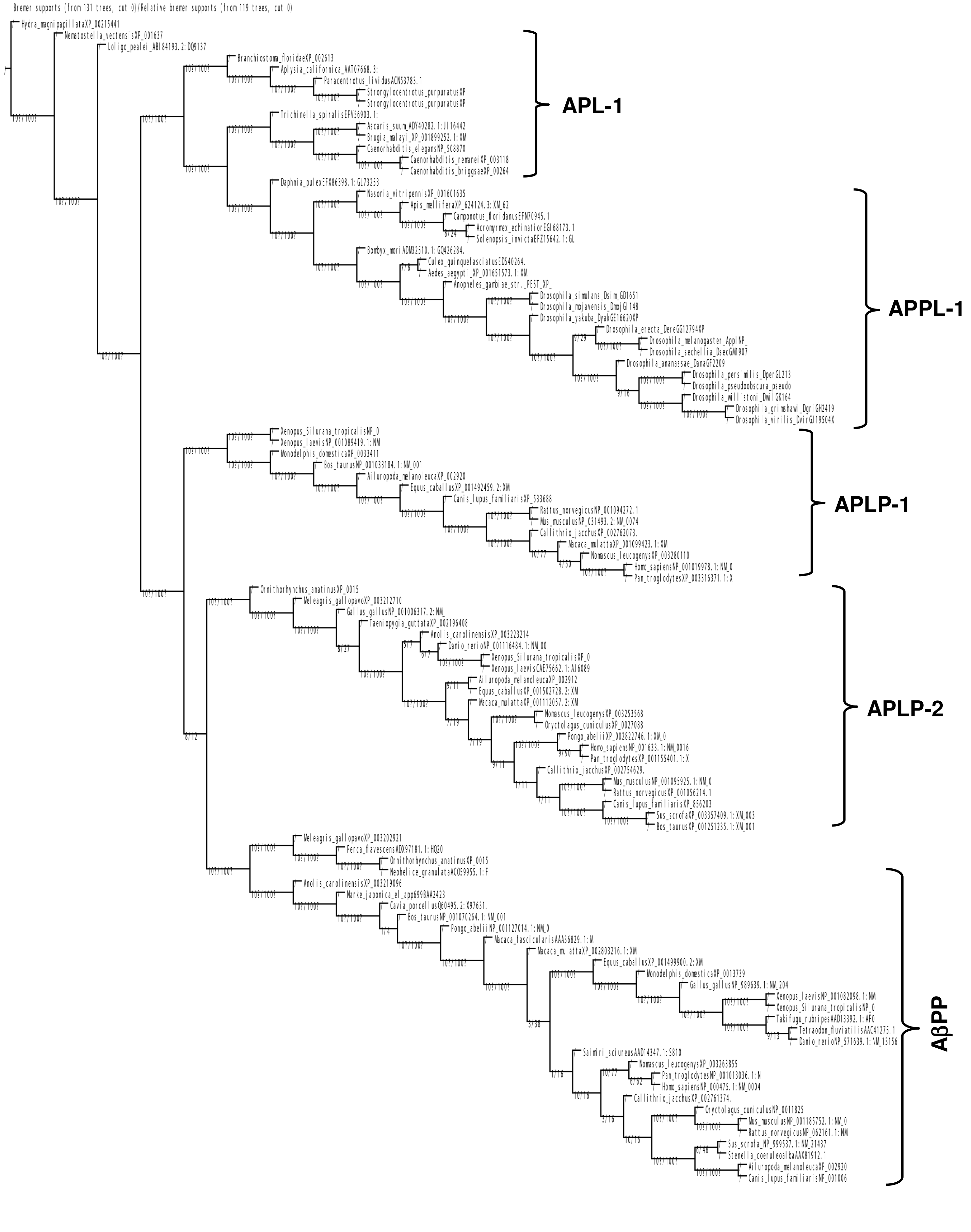

Supplementary Figure 2c

Group freqs., 100 replicates, cut=50 (tree 0) - Symmetric Resampling (P=33)/GC values, 100 replicates, cut=0 (tree 1) - Symmetric Resampling (P=33)

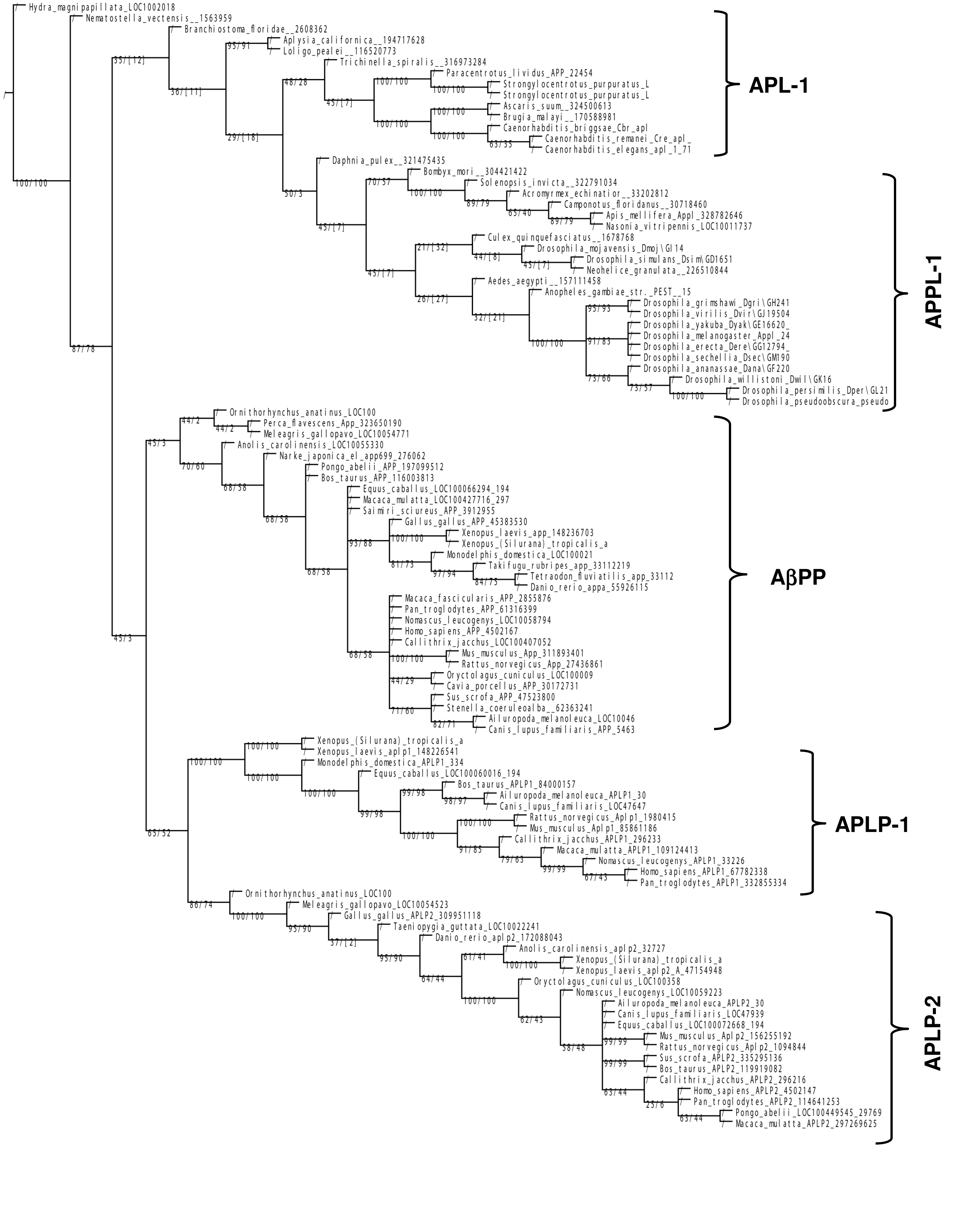

Supplementary Figure 2d

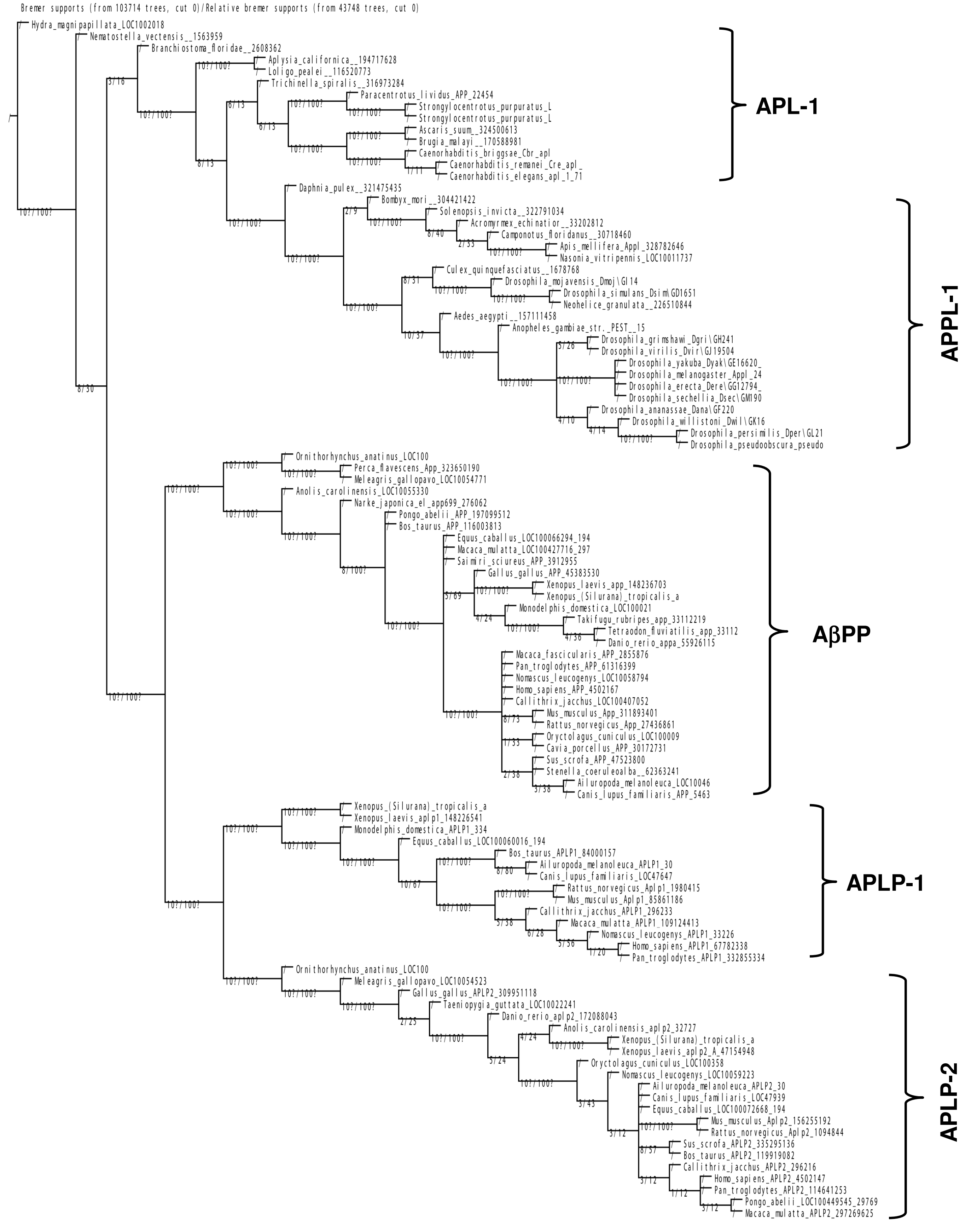

Supplementary Table 1

Taxa Species Names and Sequence Accession Numbers

Species Names	Protein Accession	Nucleotide Accession
Acromyrmex echinatior	EGI68173.1	GL888070.1
Aedes aegypti	XP 001651573.1	XM 001651523.1
Ailuropoda melanoleuca	XP 002920108.1	XM 002920062.1
Ailuropoda melanoleuca	XP 002912744.1	XM 002912698.1
Ailuropoda melanoleuca	XP 002920946.1	XM 002920900.1
Anolis carolinensis	XP 003219096.1	XM 003219048.1
Anolis carolinensis	XP 003223214.1	XM 003223166.1
Anopheles gambiae str. PEST	XP 312126.4	XM 312126.4
Apis mellifera	XP 624124.3	XM 624121.3
Aplysia californica	AAT07668.3	AY535409.3
Ascaris suum	ADY40282.1	JI164425.1
Bombyx mori	ADM32510.1	GQ426284.1
Bos taurus	NP 001070264.1	NM 001076796.1
Bos taurus	XP 001251235.1	XM 001251234.2
Bos taurus	NP 001033184.1	NM 001038095.1
Branchiostoma floridae	XP 002613121.1	XM 002613075.1
Brugia malayi	XP 001899252.1	XM 001899217.1
Caenorhabditis briggsae	XP 002644641.1	XM 002644595.1
Caenorhabditis elegans	NP 508870.3	NM 076469.4
Caenorhabditis remanei	XP 003118273.1	XM 003118225.1
Callithrix jacchus	XP 002761374.1	XM 002761328.1
Callithrix jacchus	XP 002754629.1	XM 002754583.1
Callithrix jacchus	XP 002762073.1	XM 002762027.1
Camponotus floridanus	EFN70945.1	GL437132
Canis lupus familiaris	NP 001006601.1	NM 001006601.1
Canis lupus familiaris	XP 856203.1	XM 851110.1
Canis lupus familiaris	XP 533688.2	XM 533688.2
Cavia porcellus	Q60495.2	X97631.1
Culex quinquefasciatus	EDS40264.1	DS232335.1
Danio rerio	NP 571639.1	NM 131564.1
Danio rerio	NP 001116484.1	NM 001123012.1
Daphnia pulex	EFX86398.1	GL732530.1

Supplementary Table 1 Continued

Species Names	Protein Accession	NT Accession
Drosophila ananassae Dana\GF22097	XP 001966309.1	XM 001966273.1
Drosophila erecta Dere\GG12794	XP 001982404.1	XM 001982368.1
Drosophila grimshawi Dgri\GH24196	XP 001992447.1	XM 001992411.1
Drosophila melanogaster Appl	NP 476626.2	NM 057278.3
Drosophila mojavensis Dmoj\GI14839	XP 002010177.1	XM 002010141.1
Drosophila persimilis Dper\GL21378	XP 002027785.1	XM 002027749.1
Drosophila pseudoobscura pseudoobscura Dpse\GA20547	XP 001354498.2	XM 001354462.2
Drosophila sechellia Dsec\GM19074	XP 002040243.1	XM 002040207.1
Drosophila simulans Dsim\GD16511	XP 002105827.1	XM 002105791.1
Drosophila virilis Dvir\GJ19504	XP 002055698.1	XM 002055662.1
Drosophila willistoni Dwil\GK16435	XP 002067462.1	XM 002067426.1
Drosophila yakuba Dyak\GE16620	XP 002099687.1	XM 002099651.1
Equus caballus	XP 001499900.2	XM 001499850.2
Equus caballus	XP 001502728.2	XM 001502678.2
Equus caballus	XP 001492459.2	XM 001492409.2
Gallus gallus	NP 989639.1	NM 204308.1
Gallus gallus	NP 001006317.2	NM 001006317.2
Homo sapiens	NP 000475.1	NM 000484.3
Homo sapiens	NP 001633.1	NM 001642.2
Homo sapiens	NP 001019978.1	NM 001024807.1
Hydra magnipapillata	XP 002154415.1	XM 002154379.1
Loligo pealei	ABI84193.2	DQ913735.2
Macaca fascicularis	AAA36829.1	M58727.1
Macaca mulatta	XP 002803216.1	XM 002803170.1
Macaca mulatta	XP 001112057.2	XM 001112057.2
Macaca mulatta	XP 001099423.1	XM 001099423.2
Meleagris gallopavo	XP 003202921.1	XM 003202873.1
Meleagris gallopavo	XP 003212710.1	XM 003212662.1
Monodelphis domestica	XP 001373948.2	XM 001373911.2
Monodelphis domestica	XP 003341103.1	XM 003341055.1
Mus musculus	NP 001185752.1	NM 001198823.1
Mus musculus	NP 001095925.1	NM 001102455.1

Supplementary Table 1 Continued

Species Names	Protein Accession	NT Accession
Mus musculus	NP 031493.2	NM 007467.3
Narke japonica el app699	BAA24230.1	AB005544.1
Nasonia vitripennis	XP 001601635.1	XM 001601585.1
Nematostella vectensis	XP 001637354.1	XM 001637304.1
Neohelice granulata	ACO59955.1	FJ666122.1
Nomascus leucogenys	XP 003263855.1	XM 003263807.1
Nomascus leucogenys	XP 003253568.1	XM 003253520.1
Nomascus leucogenys	XP 003280110.1	XM 003280062.1
Ornithorhynchus anatinus	XP 001506511.1	XM 001506461.1
Ornithorhynchus anatinus	XP 001519929.1	XM 001519879.1
Oryctolagus cuniculus	NP 001182598.1	NM 001195669.1
Oryctolagus cuniculus	XP 002708890.1	XM 002708844.1
Pan troglodytes	NP 001013036.1	NM 001013018.1
Pan troglodytes	XP 001155401.1	XM 001155401.2
Pan troglodytes	XP 003316371.1	XM 003316323.1
Paracentrotus lividus	ACN53783.1	FJ476254.1
Perca flavescens	ADX97181.1	HQ206583.1
Pongo abelii	NP 001127014.1	NM 001133542.1
Pongo abelii	XP 002822746.1	XM 002822700.1
Rattus norvegicus	NP 062161.1	NM 019288.1
Rattus norvegicus	XP 001056214.1	XM 001056214.2
Rattus norvegicus	NP 001094272.1	NM 001100802.1
Saimiri sciureus	AAD14347.1	S81024.1
Solenopsis invicta	EFZ15642.1	GL766058.1
Stenella coeruleoalba	AAX81912.1	AY926583.1
Strongylocentrotus purpuratus	XP 790315.2	XM 785222.2
Strongylocentrotus purpuratus	XP 001192687.1	XM 001192687.1
Sus scrofa	XP 003357409.1	XM 003357361.1
Sus scrofa	NP 999537.1	NM 214372.1
Taeniopygia guttata	XP 002196408.1	XM 002196372.1
Takifugu rubripes	AAD13392.1	AF090120.1
Tetraodon fluviatilis	AAC41275.1	AF018165.1

Supplementary Table 1 Continued

Species Names	Protein Accession	NT Accession
Trichinella spiralis	EFV56903.1	ABIR02000891.1
Xenopus (Silurana) tropicalis	NP 001005698.1	NM 001005698.1
Xenopus (Silurana) tropicalis	XP 002940052.1	XM 002940006.1
Xenopus (Silurana) tropicalis	NP 001120906.1	NM 001127434.1
Xenopus laevis	NP 001082098.1	NM 001088629.1
Xenopus laevis	CAE75662.1	AJ608932.1
Xenopus laevis	NP 001089419.1	NM 001095950.1
